# Food-derived coagulase-negative *Staphylococcus* as starter cultures for fermented foods

**DOI:** 10.1007/s10068-020-00789-5

**Published:** 2020-07-04

**Authors:** Sojeong Heo, Jong-Hoon Lee, Do-Won Jeong

**Affiliations:** 1 Department of Food and Nutrition, Dongduk Women’s University, Seoul, Republic of Korea; 2 Department of Food Science and Biotechnology, Kyonggi University, Suwon, Republic of Korea

**Keywords:** Food-derived coagulase negative staphylococci, Starter culture, Safety, Diversity

## Abstract

Food safety is of significant concern to consumers and is a major issue for the food industry. As such, the industry is aware of the importance of safety assessments of starters used in the production of fermented foods. Coagulase-negative staphylococci (CNS) are the predominant bacteria found in fermented foods worldwide. Because of this, food-derived CNS are used as starters for cheese and meat fermentation, and have been investigated for use as starters in soybean fermentation. Although food-derived CNS are generally considered non-pathogenic, their safety remains uncertain following the isolation of CNS from skin infections in humans and animals, and because they belong to the same genus as the highly pathogenic species *Staphylococcus aureus*. This review explores what is known about the safety of food-derived CNS, focusing on antibiotic resistance, enterotoxin genes, and biogenic amine production, to aid in the selection of starter candidates.

## Introduction

Staphylococci, which are identified under the microscope by the formation of irregular grape-like clusters of cells, are Gram-positive, facultatively anaerobic, catalase-positive, non-motile, and non-spore-forming bacteria with a high tolerance for salt (most strains survive in the presence of 10% NaCl) (Chapman, 1945; Götz et al., 2006). They are part of the normal microbiota of the skin and mucous membranes of both humans and animals, and are also ubiquitously distributed in a variety of niches, including soil, water, and air, as well as various foodstuffs (Coton et al., 2010b; Götz et al., 2006; Irlinger, 2008).

Staphylococci are classified as either coagulase-positive or coagulase-negative based on their ability to produce coagulase. Coagulase production is considered a virulence factor in staphylococci because it is associated with escape from phagocytic cells. Generally, coagulase-positive staphylococci (CPS) are pathogenic and/or toxigenic and are capable of causing infections such as pneumonia and food poisoning (Archer, 1998). *Staphylococcus aureus* is the representative CPS species and is a well-known virulent pathogen (Tong et al., 2015). In contrast, coagulase-negative staphylococci (CNS) are generally considered benign, despite some species (*Staphylococcus epidermidis*, *Staphylococcus haemolyticus*, and *Staphylococcus saprophyticus*) occasionally causing opportunistic infections (Kacica et al., 1994; Natoli et al., 2009; Widerstrom et al., 2012). As a result, few studies have examined the virulence of CNS species associated with fermented foods, such as *Staphylococcus carnosus*, *Staphylococcus equorum*, and *Staphylococcus xylosus*.

CNS reportedly contribute to the sensory properties of fermented foods, including color, aroma, and taste. As such, they are often used as starter cultures for meat and cheese fermentation to enhance color and flavor development (Brigante et al., 2008; Hugas and Monfort, 1997; Irlinger, 2008; Leroy et al., 2006; Schleifer et al. 2009). CNS have also been isolated from seafood- and soybean-based foods fermented at high-salt concentrations (Guan et al., 2011; Jeong et al., 2014a, 2014b), and *Staphylococcus succinus* was reported to produce species-specific volatile compounds during soybean fermentation when used as a starter culture (Jeong et al., 2017a, 2019). Despite their contribution to the sensory properties of fermented foods, CNS have not gained the qualified presumption of safety (QPS) status for use in food and feed in the European Union (EFSA, 2016). This lack of acknowledgement as food-safe microorganisms in the European Union and undocumented usage history in Korea hinders the introduction of CNS for use in fermentation in Korean food production. In this review, we explore the possibility of using CNS as starter cultures by comparing the safety of CNS species from different origins, and also examine the roles of CNS during fermentation.

## Classification, habitat, and virulence of CNS

As of 2019, the genus *Staphylococcus* comprised 53 species and 27 subspecies in the List of Prokaryotic Names with Standing in Nomenclature (Parte, 2018). Of these, 40 species and 24 subspecies were CNS, which were classified into four representative groups by colonization: *S. epidermidis* group, *Staphylococcus lugdunensis*, *S. saprophyticus* subsp. *saprophyticus*, and other CNS (Becker et al., 2014). *S. epidermidis* group species colonize human body surfaces, *S. lugdunensis* colonizes human skin, and *S. saprophyticus* colonizes the human rectum and genitourinary tract. In comparison, bacteria belonging to the other CNS species group are only found in fermented foods and used as starter cultures (Becker et al., 2014).

Becker et al. (2014) reported that *S. epidermidis*, *S. haemolyticus*, *Staphylococcus capitis*, *Staphylococcus pettenkoferi,* and *Staphylococcus auricularis* belonged to the *S. epidermidis* group, which accounts for a substantial number of foreign body-related infections and infections in preterm newborns. *S. epidermidis*-group staphylococci have been isolated from various human and animal body surfaces, both healthy and infected. Despite generally having a benign relationship with its host, *S. epidermidis* is the representative opportunistic pathogen among the CNS (Otto, 2009). Yet unlike *S. aureus*, the *S. epidermidis* toxins responsible for infection in humans have not been clearly determined (Otto, 2012). *S. haemolyticus*, *S. capitis*, and *S. pettenkoferi*, all members of the *S. epidermidis* group, have been isolated from clinical infection patients, a prosthetic joint infection, and a bacteremia case, respectively (Barros et al., 2012; Hashi et al., 2015; Tevell et al., 2017). Interestingly, approximately 55% of clinical *S. haemolyticus* isolates contain methicillin resistance genes (Barros et al., 2012), although the toxin genes remain ambiguous. Some *S. epidermidis* strains have also been shown to contain the staphylococcal cassette chromosome *mec* (SSC*mec*), coding for methicillin resistance (Otto, 2013). However, antibiotic resistance is not directly associated with virulence. Although these species have been isolated from clinical infection cases and were shown to contain virulence factors such as adhesion factors and immune effectors (Sabate Bresco et al., 2017), their direct clinical impact might be underreported. In addition, given the reports of infection caused by *S. epidermidis* group species, these bacteria may have a higher clinical burden than other CNS.

*Staphylococcus lugdunensis*, a normal inhabitant of human skin, was first described by Freney et al. (1988) and has never been isolated from fermented foods. *S. lugdunensis*-associated skin infections have been reported, causing symptoms similar to those of *S. aureus* infection (Bieber and Kahlmeter, 2010). However, it is not clear what virulence factors *S. lugdunensis* uses for infection. In Greece, *S. lugdunensis* was isolated from various infection sites in hospitalized patients (Giormezis et al., 2015). Bierowiec et al. reported that while most tested *S. lugdunensis* isolates were susceptible to nine antibiotics (penicillin G, gentamicin, rifampicin, cefoxitin, fusidic acid, trimethoprim/sulfamethoxazole, norfloxacin, clindamycin, and erythromycin), some showed resistance to erythromycin and/or tetracycline via strain-specific carriage of *ermC* and/or *tetK* (Bierowiec et al., 2019). In addition, a very small number of *S. lugdunensis* isolates carried SSC*mec* type IVa. In contrast, a survey of clinical *S. lugdunensis* isolates by Giormezis et al. (2015) found high rates of resistance to ampicillin (50%), erythromycin (18.4%), and clindamycin (18.4%).

*Staphylococcus saprophyticus* subsp. *saprophyticus* is frequently isolated from young female outpatients with uncomplicated urinary tract infections but is rarely detected in healthy individuals, and has occasionally been implicated in cases of pyelonephritis, septicemia, nephrolithiasis, and endocarditis (Raz et al., 2005). Genome analysis revealed that *S. saprophyticus* ATCC 15305^T^ does not possess genes coding for common *S. aureus* virulence factors such as coagulase and enterotoxins but does contain genes responsible for urease production and adhesion to uroepithelial cells, which might be involved in establishing uncomplicated urinary tract infections (Kuroda et al., 2005). While *S. saprophyticus* surface-associated protein and lipase have been suggested as potential virulence factors (Sakinc et al., 2005, 2007; Szabados et al., 2013; Tang et al., 2009), no investigations into the pathogenicity of *S. saprophyticus* in the urinary tract or the expression of potential virulence genes in this environment have been published as yet.

*Staphylococcus saprophyticus* is also commonly detected in fermented foods such as Greek and Italian fermented sausages (Drosinos et al., 2005; Mauriello et al., 2004; Papamanoli et al., 2002), Taiwanese naturally-fermented dry ham (Tu et al., 2010), and Korean fermented soybeans (Jeong et al., 2014b). It was also associated with the improvement of sausage aromatization during ripening (Mauriello et al., 2004; Samelis et al., 1998). *Staphylococcus saprophyticus* has been isolated and assessed for use as a starter culture for fermentation of French and Spanish dry sausages (Fonseca et al., 2013; Montel et al., 1993), as well as Korean fermented soybeans (Jeong et al., 2016a). Multilocus sequence typing (MLST)-based analysis of *S. saprophyticus* subsp. *saprophyticus* strains from fermented foods and clinical specimens revealed a correlation between the genetic backgrounds of the strains and their origins, and identified a link between genetic background and acquisition of lincomycin resistance (Lee et al., 2015). The results suggested that functional properties such as virulence and antimicrobial susceptibility might differ in an origin-dependent manner among *S. saprophyticus* subsp. *saprophyticus* strains. However, the notoriety of *S. saprophyticus* as an uropathogen hinders its application as a starter for fermented foods despite its documented contribution to the sensory properties of various foods during fermentation. Therefore, more evidence is needed to confirm the origin-dependence of the genetic backgrounds of *S. saprophyticus* strains before they can be used in food fermentation.

Other CNS classified by Becker et al. include *S. carnosus*, *Staphylococcus condimenti*, *S. equorum*, *Staphylococcus piscifermentans*, *S. succinus*, and *S. xylosus* (Becker et al., 2014), all of which are typically associated with fermented foods (Irlinger, 2008). Irlinger suggested that food-derived CNS from milk or dairy products should be classified as exceptional opportunistic pathogens (Irlinger, 2008) because although they are primarily nonpathogenic, there are isolated reports of these CNS species being recovered from clinical samples (Blaiotta et al., 2004; Novakova et al., 2006). Coton et al. (2010a) investigated 297 clinical CNS isolates and showed that they predominantly belonged to the species *S. epidermidis*, *S. capitis*, *Staphylococcus hominis*, *Staphylococcus warneri*, and *S. haemolyticus*, and none were identified as food-associated species *S. carnosus, S. condimenti*, *S. equorum*, *S. piscifermentans*, *S. succinus*, or *S. xylosus*. However, the relationship between food-derived CNS and human infection is still unclear.

## Safety of CNS derived from fermented foods

The Food Safety Authority of the European Union has introduced the QPS approach for safety assessment of microorganisms throughout the food chain, with emphasis on acquired antibiotic resistance, presence of toxin genes (including hemolysin genes), and production of biogenic amines (EFSA, 2004). Four species, *S. carnosus*, *S. equorum*, *S. succinus*, and *S. xylosus*, account for the majority of food-derived CNS isolates from fermented meat, sausage, soybean products, and fish sauce (Becker et al., 2014; Guan et al., 2011; Jeong et al., 2014b; Mauriello et al., 2004; Sondergaard and Stahnke, 2002). As such, several studies including safety assessments of these four CNS species using the QPS approach have been undertaken.

### *Staphylococcus carnosus*

*Staphylococcus carnosus* has been used as a starter culture for sausage fermentation since the 1950s (Lofblom et al., 2017). This long usage history has shown it to be a benign food-grade species, with complete genome sequencing of *S. carnosus* TM300 revealing that it lacks genes required for pathogenicity (Rosenstein et al., 2009).

Most *S. carnosus* isolates from various fermented foods are sensitive to antibiotics; however, a few strains have shown resistance to a small number of antibiotics (Martin et al., 2006; Marty et al., 2012; Muller et al., 2016; Resch et al., 2008) (Table 1). For example, Muller et al. (2016) studied the antibiotic resistance profiles of 39 *S. carnosus* strains isolated from fermented sausage meat and found that while all 39 strains were susceptible to ampicillin, amoxicillin/clavulanic acid, ciprofloxacin, clindamycin, erythromycin, gentamicin, imipenem, kanamycin, linezolid, quinupristin/dalfopristin, rifampicin, tetracycline, and vancomycin), four and two strains showed resistance or intermediate resistance to chloramphenicol and oxacillin, respectively. Similarly, Marty et al. (2012) showed that while all 21 *S. carnosus* strains from fermented meats were susceptible to ampicillin, amoxicillin, clindamycin, cloxacillin, erythromycin, fusidic acid, methicillin, oxacillin, penicillin G, and tetracycline, 14% of the strains were resistant to streptomycin and trimethoprim (Marty et al., 2012). Interestingly, and unusually for *S. carnosus* strains, another study showed that while all tested *S. carnosus* strains from fermented sausages were sensitive to 12 different antibiotics, all 11 strains were resistant to ampicillin (Martin et al., 2006). Together, these findings suggest that *S. carnosus* has low overall rates of antibiotic resistance, and that any antibiotic resistance that does occur appears to be strain specific.

**Table 1:**
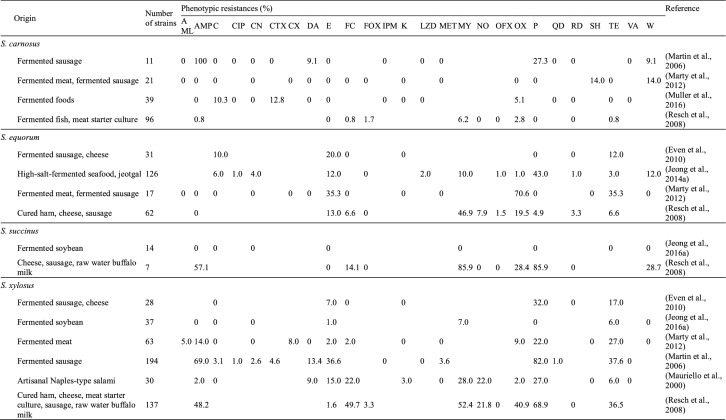
Antibiotic resistance profiles of food-derived coagulase-negative staphylococci

Blank box indicates that the antibiotic was not assessed

Antibiotics: AML, amoxicillin; AMP, ampicillin; C, chloramphenicol; CIP, ciprofloxacin; CN, gentamicin; CTX, cefotaxime; CX, cloxacillin; DA, clindamycin; E, erythromycin; FC, fusidic acid; FOX, cefoxitin; IPM, imipenem; K, kanamycin; LZD, linezolid; MET, methicillin; MY, lincomycin; NO, novobiocin; OFX, ofloxacin; OX, oxacillin; P, penicillin G; QD, quinupristin/dalfopristin; RD, rifampin; SH, streptomycin; TE, tetracycline; VA, vancomycin; W, trimethoprim

Staphylococcal enterotoxin genes, exfoliative toxin genes, and the toxic shock syndrome toxin-encoding gene have not been amplified from any of the *S. carnosus* strains examined to date (Table 2) (Martin et al., 2006; Muller et al., 2016). Only one among the 39 strains examined by Muller et al. in 2016 showed weak hemolysis activity (Muller et al., 2016), while a hemolysin gene was amplified from a second strain. Thus, the phenotype and genotype did not match for either strain. To date, there have been several reports of *S. carnosus* isolates showing hemolysis on solid media and successful PCR-based amplification of a partial hemolysin gene from some strains; however, the in vitro and in vivo virulence of *S. carnosus* has not been reported (Muller et al., 2016). Importantly though, the genome sequence of *S. carnosus* TM300 lacks virulence genes encoding the enterotoxin and hemolysin (Rosenstein et al., 2009).

**Table 2:** Prevalence of hemolysis, hemolysin, and toxin genes among food-derived coagulase-negative staphylococci

	*S. carnosus*			*S. equorum*			*S. succinus*	*S. xylosus*		
Hemolysis										
Horse blood						0	0			11.1
Sheep blood						2.4	0			16.7
Human blood		60.0	2.6	88.2				3.0		
Hemolysin genes										
*hla*		0	2.6	0	0	0			0	
*hlb*		0	0	0	0	0			0	
*hld*		0	2.6	23.5	0	0		0	3.6	
SE_0613		0		0	3.2	0		0	0	
SE_1760		0		0	3.2	0		0	0	
Enterotoxin genes										
*sea*	0	0	0	0		0		0	0	
*seb*	0	0	0	0	0	0		0	0	
*sec*	0	0	0	0		0		1.5	0	
*sed*	0	0	0	0	0	0		0	0	
*see*	0	0	0	0	0	0		0	0	
*seg*		0		5.9					0	
*seh0*		0		0					0	
*sei*		0		0					0	
*sej*				5.9	0				0	
*selj*					0				0	
*selr*					0				0	
*set9*					0				0	
Exfoliative toxin genes										
*eta*		0	0	0	0				0	
*etb*		0		0	0				0	
Toxic shock syndrome toxin gene										
*tst*-*1*		0	0	0	0				0	
Number of strains	11	5	39	17	31	126	14	194	28	18
References	Martin et al. (2006)	Marty et al. (2012)	Muller et al. (2016)	Marty et al. (2012)	Even et al. (2010)	Jeong et al. (2014a)	Jeong et al. (2016a)	Martin et al. (2006)	Even et al. (2010)	Jeong et al. (2016a)

Blank box indicates an assay was not performed

SE_0613 and SE_1760 are the hemolysin- and hemolysin III-encoding genes, respectively, from *S. epidermidis* ATCC 12228

*hylIII,* hemolysin III-encoding gene from *Bacillus cereus*

*Staphylococcus carnosus* does not produce the biogenic amines cadaverine, putrescine, tryptamine, tyramine, or histamine. However, some strains do produce phenethylamine, which is derived from phenylalanine via decarboxylation (Table 3) (Martin et al., 2006; Muller et al., 2016), although the amounts produced, even with an abundance of the precursor amino acid, are below the allowable concentration listed in Codex (100–200 μg/mL). In addition, the phenylalanine decarboxylase gene, which is required for phenethylamine production, was not detected in the genome of *S. carnosus* TM300 (Rosenstein et al., 2009).

**Table 3:** Prevalence of in vitro production of biogenic amines and associated genes in coagulase-negative staphylococci

	Biogenic amines												Number of strains	Method	References
	Cadaverine		Histamine		Phenyl-ethylamine		Putrescine		Tryptamine		Tyramine				
	P^a^	G^b^	P^a^	G^b^	P^a^	G^b^	P^a^	G^b^	P^a^	G^b^	P^a^	G^b^			
*S. carnosus*															
	0		0		90.9		0		0		0		11	HPLC	Martin et al. (2006)
	0		0		63.2		0						39	HPLC	Muller et al. (2016)
*S. equorum*															
	–	3.2	–	0	(116 μg/mL)^c^	0			(23 μg/mL)^c^	0			31	LC	Even et al. (2010)
	(29.6 ppm) ^d^		(40.0 ppm) ^d^				(22.6 ppm) ^d^				(29.7 ppm) ^d^		39	HPLC	Jeong et al. (2014a)
		85.7		0		14.3		0		0		0	7	Medium	Bonomo et al. (2009)
*S. succinus*															
		100				0		0		0	0		2	Medium	Bonomo et al. (2009)
	76.2		0		32		76.2		41.3			10.5	9	HPLC	Jeong et al. (2016a)
*S. xylosus*															
										49.0	49.0		51	HPLC	Martuscelli et al. (2000)
	1		0.5		6.7		1.5			0.5	3.6		194	HPLC	Martin et al. (2006)
		0		0		0			0			0	28	LC	Even et al. (2010)
		41.2				5.9		0	5.9			0	17	Medium	Bonomo et al. (2009)
	72.1		0		32.8		72.1			42.8	11		2	HPLC	Jeong et al. (2016a)

Blank box indicates an assay was not conducted

–Concentration below the limit of detection

^a^Phenotype

^b^Genotype

^c^Biogenic amines produced by only 1 out of 31strains

^d^Concentration of biogenic amines produced by the highest producers

### *Staphylococcus equorum*

*Staphylococcus equorum* is a common component of the microbial communities of high-salt-fermented foods produced in Europe such as meat products and smear-ripened and semi-hard cheeses (Blaiotta et al., 2004; Bockelmann et al., 2005; Corbiere Morot-Bizot et al., 2006; Mauriello et al., 2004; Place et al., 2003). In addition, *S. equorum* has been developed as a starter culture for fermented foods such as sausage and jeotgal (Jeong et al., 2014a; Mauriello et al., 2004; Place et al., 2003; Sondergaard and Stahnke, 2002). The genome sequence of *S. equorum* Mu2 from a French smear-ripened cheese revealed that it does not contain any of the virulence factors found in *S. aureus* (Irlinger et al., 2012), and genomic analysis of *S. equorum* KS1039, which has been selected as a starter candidate for jeotgal, confirmed that it is also missing the *S. aureus* virulence genes (Jeong et al., 2016b).

In a study by Even et al. (2010), all 31 tested *S. equorum* strains were susceptible to fusidic acid, kanamycin, penicillin G, and rifampicin, with fewer than 20% of strains found to be resistant to chloramphenicol, erythromycin, and tetracycline (Table 1). Marty et al. (2012) reported similar findings, with 17 strains isolated from fermented meats showing susceptibility to ampicillin, amoxicillin, chloramphenicol, clindamycin, cloxacillin, gentamycin, fusidic acid, kanamycin, methicillin, penicillin G, streptomycin, and trimethoprim, and approximately 20% of strains showing resistance to erythromycin, oxacillin, and tetracycline. In comparison, Resch et al. (2008) found that most *S. equorum* isolates were resistant to at least one antibiotic, with 46.9% of the tested isolates demonstrating resistance to lincomycin. As well as being common in fermented foods in Europe, *S. equorum* has also been identified as the dominant microbial species in jeotgal, a high-salt-fermented seafood product from Korea (Guan et al., 2011; Jeong et al., 2014a). One study found that 66/126 ampicillin-sensitive *S. equorum* strains from jeotgal exhibited phenotypic resistance to at least one antibiotic, with the highest rates of resistance observed for penicillin G (34.1%), erythromycin (9.5%), and trimethoprim (9.5%). Therefore, among the food-derived CNS, the antibiotic resistance of *S. equorum* appears to be unrivaled. Comparative genomic analysis of six *S. equorum* strains revealed the presence of several antibiotic resistance genes, although four of the strains were sensitive to the corresponding antibiotics (Jeong et al., 2017a). In the two remaining strains, antibiotic resistance was conferred by acquired antibiotic resistance genes.

Safety assessments of 17 *S. equorum* isolates from spontaneously-fermented Swiss meat products showed no antibiotic resistance and produced negative results for other safety concerns such as hemolysis, hemagglutination, virulence factors, and biogenic amines (Marty et al., 2012) (Table 2). In addition, while staphylococcal enterotoxin genes were not present in most of the isolates, partial *seg* and *sej* gene sequences were amplified from one isolate (Table 2) (Marty et al., 2012). Further screening of *S. equorum* isolates from jeotgal (*n* = 126) and fermented sausages and cheese (*n* = 31) also confirmed the absence of staphylococcal enterotoxin genes (Even et al., 2010; Jeong et al., 2014a). However, a complete enterotoxin gene sequence and the contribution of enterotoxins to virulence in *S. equorum* have not been reported. Weak hemolysis and moderate hemolytic activity were frequently observed among *S. equorum* isolates in a study by Marty et al. (2012), and a partial δ-hemolysin-encoding gene (*hld*) was amplified from one isolate. Similarly, *S. equorum* isolates from jeotgal commonly demonstrated δ-hemolysis, although α- and β-hemolysis were infrequently detected (Jeong et al., 2014a). Comparative genomic analysis of six *S. equorum* strains failed to identify α-, β-, and δ-hemolysin genes with homology to those observed in *S. aureus* (Jeong et al., 2017b), although several other annotated hemolysin genes were present. Interestingly, these other hemolysin genes are often found in non-hemolytic *S. equorum* strains, while hemolytic strains show strain-specific carriage of a gene encoding a hemolysin activation protein and a hemolysin-family calcium-binding region (Jeong et al., 2017b). These results suggest that *S. equorum* does not generally carry enterotoxin or hemolysin genes but some specific strains may have acquired them.

Most *S. equorum* isolates from traditionally-fermented sausages produced in the Basilicata region of Italy tested positive for decarboxylation of lysine and phenylalanine based on a color change on indicator media (Bonomo et al., 2009). However, 78% of the isolates did not carry any genes involved in biogenic amine production (Even et al., 2010). High-performance liquid chromatography (HPLC) analysis showed that some *S. equorum* isolates from jeotgal produced low concentrations of cadaverine, histamine, putrescine, and tyramine in media supplemented with amino acid precursors (Table 3)(Jeong et al., 2014a). However, comparative genomic analysis of six different strains revealed that while the *S. equorum* strains carried genes involved in cadaverine production, none of the genes required for production of histamine, putrescine, or tyramine were present in any of the genomes (Lee et al., 2018). These results confirm that *S. equorum* is fundamentally a benign species.

### *Staphylococcus succinus*

*Staphylococcus succinus* is one of the predominant bacterial species in fermented sausage (Corbiere Morot-Bizot et al., 2006; Mauriello et al., 2004; Talon et al., 2008) and is used as a starter culture in various meat fermentation processes (Talon et al., 2008). Resch et al. (2008) found that *S. succinus* has high levels of antibiotic resistance against ampicillin, lincomycin, and penicillin G. However, safety assessments of 81 CNS isolates from fermented soybeans showed that none of the *S. succinus* isolates exhibited antibiotic resistance or hemolytic activity (Jeong et al., 2016a). Among them, strain 14BME20 was selected as a starter candidate. Genomic analysis confirmed that strain 14BME20 did not contain any of the known *S. aureus* virulence factor-encoding genes but did carry strain-specific genes for lipid degradation, which may contribute to the production of volatile compounds (Jeong and Lee, 2017).

*Staphylococcus succinus* isolates from doenjang, a high-salt-fermented soybean product, did not exhibit hemolysis (Jeong et al., 2016a) and attempts to amplify enterotoxin genes were unsuccessful (unpublished results). However, the isolates did produce cadaverine, putrescine, and tyramine, but not histamine, in a laboratory setting (Jeong et al., 2016a). Additionally, genome sequencing of the *S. succinus* isolates suggested that the genes required for histamine, putrescine, and tyramine production were missing from all isolates, although a lysine decarboxylase-encoding gene, which is needed for cadaverine production, was identified (Jeong and Lee, 2017; Megaw and Gilmore, 2016; Zhou et al., 2017). Cadaverine production by the isolates averaged 75.1 ppm in medium supplemented with excess precursors, suggesting that if precursor concentrations were restricted, cadaverine production should be very low.

### *Staphylococcus xylosus*

Like *S. succinus*, *S. xylosus* is a predominant species in fermented sausages (Blaiotta et al., 2004; Corbiere Morot-Bizot et al., 2006; Mauriello et al., 2004; Talon et al., 2008) and is used as a starter culture in fermented sausage production because of its contribution to color and flavor development (Fiorentini et al., 2009; Martin et al., 2006; Talon et al., 2008). Genome sequence analysis of a *S. xylosus* strains used as a meat starter culture revealed that all known *S. aureus* genes coding for enterotoxins and virulence factors were missing from the genome (Labrie et al., 2014).

However, *S. xylosus* isolates have shown high levels of resistance to ampicillin, penicillin G, and tetracycline (Even et al., 2010; Martin et al., 2006; Marty et al., 2012; Mauriello et al., 2000; Resch et al., 2008), although the *mecA* gene, which is responsible for methicillin resistance, has only rarely been identified in *S. xylosus* (Martin et al., 2006; Resch et al., 2008). Microarray analysis of several *S. xylosus* isolates by Kastner et al. (2006) showed that while most antibiotic resistance genes were not present, partial *tetK* genes, responsible for tetracycline resistance, were detected in all of the tested isolates. However, other studies have not detected the *tetK* gene in *S. xylosus*. *S. xylosus* is a predominant species in fermented soybean along with *S. succinus* and *S. saprophyticus* (Jeong et al., 2014b, 2016a). A study by Jeong et al. (2014b) revealed that all 37 examined *S. xylosus* isolates from doenjang were susceptible to ampicillin, chloramphenicol, gentamycin, penicillin G, and trimethoprim, although 7% of isolates were resistant to erythromycin, lincomycin, and streptomycin.

Safety assessments of 18 *S. xylosus* isolates from doenjang showed that two and three isolates displayed weak α-hemolytic and β-hemolytic activity, respectively. However, α-hemolysin and β-hemolysin genes were not detected in the genome sequences of the *S. xylosus* isolates (Labrie et al., 2014). Similarly, approximately 3% of *S. xylosus* isolates from fermented sausages in a study by Martin et al. (2006) showed δ-hemolysis activity despite none of the isolates containing *hld* (Table 2). Amplification of staphylococcal enterotoxin genes has also been unsuccessful from most *S. xylosus* isolates (Table 2) (Even et al., 2010; Martin et al., 2006).

According to Ansorena et al. (2002), biogenic amines 2-phenylethylamine, putrescine, histamine, and tryptamine were not detected in sausages produced using a *S. xylosus* starter, although the *S. carnosus* starter cultures showed production of 2-phenylethylamine and tryptamine. Martuscellin et al. reported that approximately 80% of *S. xylosus* strains from artisanal fermented sausages did not demonstrate amino acid decarboxylase activity, which is required for biogenic amine production from amino acids, and 52% of strains were not able to produce biogenic amines (Martuscelli et al., 2000). Therefore, the production of biogenic amines by food-derived CNS appears to strain-specific (Table 3) (Ansorena et al., 2002; Bonomo et al., 2009; Even et al., 2010; Jeong et al., 2014a; Martin et al., 2006; Martuscelli et al., 2000). Additionally, genome sequence analysis of several *S. carnosus*, *S. equorum*, *S. succinus*, and *S. xylosus* isolates showed that none of the genomes contained genes involved in histamine and tyramine production; however, a lysine decarboxylase-encoding gene, which is required for cadaverine production, was identified (Table 4). These results again suggest that biogenic amines production might be strain-specific in *S. xylosus*.

**Table 4:** Potential genes associated with biogenic amine production in five food-derived coagulase-negative staphylococci

Product	*S. carnosus*	*S. equorum*	*S. succinus*	*S. xylosus*	*S. saprophyticus*
	TM300	KS1039	14BME20	C2a	ATCC 15305
Lysine decarboxylase	SCA_RS00595	SE1039_RS01180	BK815_RS08125	SXYL_RS11985	SSP_RS11450
	SCA_RS01670	SE1039_RS02575	BK815_RS06925	SXYL_RS10610	SSP_RS10205

Food-derived CNS species are occasionally isolated from human skin infections (Blaiotta et al., 2004; Novakova et al., 2006). However, direct evidence of the involvement of food-derived CNS species in human disease is insufficient, and the virulence mechanism has yet to be determined. Moreover, genomic analysis revealed that food-derived CNS do not produce the adhesion proteins, invasion proteins, and enterotoxins that are necessary for the virulence of *S. aureus* (Heo et al., 2020; Jeong et al., 2017b). Therefore, we suggest that food-derived CNS are likely to be suitable for use in food production.

## Contribution of food-derived CNS to food fermentation

CNS contribute to the sensory qualities of sausages via the catabolism of carbohydrates and amino acids, the formation of esters, and their interaction with fatty acids (Montel et al., 1998). CNS produce small molecule flavor compounds through proteolysis and lipolysis reactions (Berdague et al., 1993; Stahnke, 1994; Sondergaard and Stahnke, 2002). For example, *S. equorum* from jeotgal exhibited protease and lipase activity, while *S. saprophyticus*, *S. succinus*, and *S. xylosus* from doenjang showed protease and lipase activity (Jeong et al., 2014a, 2016a). During in vitro fermentation, *S. carnosus*, *S. equorum*, and *S. xylosus* were responsible for different aroma profiles, with *S. carnosus* producing higher concentrations of leucine-, isoleucine-, and valine-derived volatile compounds (Sondergaard and Stahnke, 2002). *S. equorum* from surface-ripened French cheese produced volatile compounds such as 3-methyl-3-buten-1-ol and 4-methyl-2-pentanone, which are responsible for fruity and sweet descriptors (Deetae et al., 2007), while *S. succinus* produced 3-mehylbutyl acetate, which is responsible for banana and pear descriptors, during soybean fermentation (Jeong et al., 2017a, 2019). CNS also contributed the volatile profile during fermentation of dry sausages (Ravyts et al., 2010). Additionally, *S. succinus* affected the volatile compounds detected in fermented soybean during pilot plant fermentation. These volatile compounds were derived from the conversion of amino acids (Jeong et al., 2019). Jeong et al. (2017a) also confirmed that the profile of volatile compounds produced by *S. succinus* was different from that of *S. saprophyticus* during soybean fermentation.

Unlike most lactic acid bacteria, genome sequence analysis of six strains revealed that *S. equorum* contains genes for the biosynthesis of all amino acids except asparagine and the conversion of branched chain fatty acids (Lee et al., 2018). *S. equorum* also carries genes required for the production of butane-2,3-diol, diacetyl, and acetoin via glycolysis, and ester compounds via protein degradation (Lee et al., 2018). In fermented sausage, *S. succinus* and *S. xylosus* produced acetoin diacetyl (Ravyts et al., 2010), while Flores and Toldra (2011) showed that CNS enzymatically converted free amino acids from peptides. Together, these results show that CNS contribute to flavor development in fermented foods via carbohydrate fermentation, proteolysis, lipolysis, and amino acid conversions. CNS can therefore be selected based on these various properties for use as starter cultures for fermentation.

## Multilocus sequence typing and diversity of food-derived CNS

Food-derived CNS have several advantages for use as potential starter cultures for meat, seafood, and soybean fermentation. However, they also belong to the same genus as important human pathogen *S. aureus*, and have occasionally been isolation from human skin infections. In addition, food-derived CNS are still not included on the Pan American Health Organization list of safe organisms, despite the assertions of Irlinger (2008) that no CNS isolated from milk or dairy products has ever been involved in case of food poisoning or human pathology. Therefore, genetic and phenotypic differences between food-derived and clinical CNS isolates needs to be explored, along with the genetic diversity among food-derived CNS.

MLST based on internal fragments of five to seven housekeeping genes is an efficient tool for characterizing bacterial species in short-term epidemiological studies. As such, MLST has been used for strain characterization of food-derived CNS including *S. carnosus* (Buckle et al., 2017), *S. equorum* (Jeong et al., 2014c), and *S. saprophyticus* (Lee et al., 2015).

To examine the genetic diversity and population structure of *S. carnosus* strains, MLST using seven housekeeping genes (*glpK* (encoding glycerol kinase), *tpiA* (triosephosphate isomerase), *dat* (D-amino acid aminotransferase), *xprT* (xanthine phosphoribosyltransferase), *gmk* (guanylate kinase), *narG* (respiratory nitrate reductase alpha chain), and *cstA* (carbon starvation protein)) was conducted (Buckle et al., 2017). A total of 44 *S. carnosus* isolates from fish sauce, fish brine, sausage, smoked raw ham, salami, beef, and starter cultures were examined using the MLST assays. The results showed no correlation between sequence type and/or isolation year or source.

A MLST scheme was developed for *S. saprophyticus* to evaluate the genetic diversity between isolates from food and clinical origins based on seven housekeeping genes: *aroE* (encoding shikimate 5-dehydrogenase), *dnaJ* (chaperone protein), *glpF* (glycerol 3-phosphate dehydrogenase), *gmk* (guanylate kinase), *hsp60* (heat shock protein 60), *mutS* (DNA mismatch repair protein), and *pta* (phosphotransacetylase) (Lee et al., 2015). A total of 48 isolates from human urine (*n* = 21), doenjang (*n* = 9), myeolchi-jeotgal (*n* = 6), saeu-jeotgal (*n* = 3), and sausage (*n* = 9) samples were subjected to MLST. Again, the results showed no correlation between sequence type and isolate origin, but clustering of the sequence types using the eBURST algorithm (Feil et al., 2004) revealed a correlation between the genetic backgrounds and origins of the isolates.

Another study developed a MLST scheme to evaluate the genetic diversity and background of *S. equorum* based on seven housekeeping genes: *aroE*, *dnaJ*, *glpF*, *gmk*, *hsp60*, *mutS*, and *pta* (Jeong et al., 2014c). A total of 117 *S. equorum* strains from saeu-jeotgal, myeolchi-jeotgal, sausage, cheese, and horse skin were examined. Results confirmed that MLST could not accurately identify correlations between the origins of the isolates and their sequence types.

Therefore, there is little information that can be used to accurately discriminate differences between CNS isolates of food and clinical origin. Complicating the issue is the lack of clinical isolates given the very rare occurrence of food-derived CNS-associated human infection. Additionally, CNS identification using commercial identification kits and ribotyping is difficult because of the variability within species (Carretto et al., 2005; Dupont et al., 2010; Sivadon et al., 2004), increasing the possibility of CNS misidentification. In addition, there is no consensus on the identification methods used. For example, *S. xylosus* isolates from patients with urinary tract infections were identified using phenotypic analysis and API 20 staph strips (Al-Mathkhury et al., 2008), while *S. xylosus* from bacteremia patients was identified using phenotypic methods only (Tselenis-Kotsowilis et al., 1982). Therefore, we suggest that species identification should be confirmed using several methods, especially food-derived CNS clinical isolates.

## Food-derived CNS as starter candidates

Fermented foods are reservoirs and vehicles for large populations of living bacteria; therefore, the safety of starter cultures has become a significant issue. Prospective starters must be assessed for safety as well as functional properties such as the enhancement of sensory properties including color and volatile compound improvement. The safety of a starter is mainly assessed by two approaches: the Generally Recognized As Safe (GRAS) notification system in the United States and QPS in the European Union. The QPS system is based on four pillars of safety assessment dealing with taxonomy (establishing identity), familiarity (history of use, scientific literature, clinical aspects, industrial application, and ecology), pathogenicity (identification of safety concerns), and end use. Among the food-derived CNS, *S. carnosus* and *S. xylosus* have been nominated to achieve QPS status because of their extensive usage history in traditional meat fermentation (Talon and Leroy, 2011). However, further research is needed to obtain sufficient knowledge about the safety of food-derived CNS before they obtain QPS status. Food-derived CNS require case-by-case safety evaluations before they are cleared for use as starter cultures.

This review highlights the remaining safety concerns surrounding the use of food-derived CNS as starter cultures, particularly the small number of clinical infection cases attributed to these species. However, there have been no reported cases of food poisoning caused by food-derived CNS. In addition, comparative genomic analysis revealed that food-derived CNS do not carry the genes required for histamine, tyramine, putrescine, or cadaverine production. In terms of safety, the main hazard of bacteria used in food fermentation is their ability to acquire antibiotic resistance determinants, rather than their intrinsic resistance (EFSA, 2005). Intrinsic resistance is conferred by chromosomally-encoded genes that have been altered by mutation, and there is a very low risk of horizontal transmission of these genes. In contrast, acquired antibiotic resistance genes are located on mobile elements, which are readily transferred among strains. Given the lack of evidence of acquired antibiotic resistance genes and virulence genes in food-derived CNS, we propose that food-derived *S. carnosus*, *S. equorum*, *S, succinus*, and *S. xylosus* strains should be recognized as safe for use as starter cultures in food fermentation and they are should be contributed the enhancement of sensory properties including color, flavor, and taste in fermented food.
