# Correction to: Food-derived coagulase-negative *Staphylococcus* as starter cultures for fermented foods

**DOI:** 10.1007/s10068-020-00806-7

**Published:** 2020-09-05

**Authors:** Sojeong Heo, Jong-Hoon Lee, Do-Won Jeong

**Affiliations:** 1 Department of Food and Nutrition, Dongduk Women’s University, Seoul, Republic of Korea; 2 Department of Food Science and Biotechnology, Kyonggi University, Suwon, Republic of Korea

## Correction to: Food Sci Biotechnol (2020) 29(8):1023–1035 10.1007/s10068-020-00789-5

The article “Food-derived coagulase-negative *Staphylococcus* as starter cultures for fermented foods”, written by Sojeong Heo, Jong-Hoon Lee, and Do-Won Jeong, was originally published Online First without Open Access. After publication in volume 29, issue 8, page 1023–1035 the author decided to opt for Open Choice and to make the article an Open Access publication. Therefore, the copyright of the article has been changed to © The Author(s) 2020 and the article is forthwith distributed under the terms of the Creative Commons Attribution 4.0 International License (http://creativecommons.org/licenses/by/4.0/), which permits use, duplication, adaptation, distribution and reproduction in any medium or format, as long as you give appropriate credit to the original author(s) and the source, provide a link to the Creative Commons license, and indicate if changes were made.

The original article has been corrected.

